# Single-cell transcriptome of bronchoalveolar lavage fluid reveals sequential change of macrophages during SARS-CoV-2 infection in ferrets

**DOI:** 10.1038/s41467-021-24807-0

**Published:** 2021-07-27

**Authors:** Jeong Seok Lee, June-Young Koh, Kijong Yi, Young-Il Kim, Su-Jin Park, Eun-Ha Kim, Se-Mi Kim, Sung Ho Park, Young Seok Ju, Young Ki Choi, Su-Hyung Park

**Affiliations:** 1 GENOME INSIGHT Inc., Daejeon, Republic of Korea; 2 Graduate School of Medical Science and Engineering, Korea Advanced Institute of Science and Technology (KAIST), Daejeon, Republic of Korea; 3 College of Medicine and Medical Research Institute, Chungbuk National University, Cheongju, Republic of Korea; 4 Division of Life Science, Research Institute of Life Science, Gyeongsang National University, Jinju, Korea; 5 School of Life Sciences, Ulsan National Institute of Science & Technology (UNIST), Ulsan, Republic of Korea; 6 The Center for Epidemic Preparedness, KAIST Institute, KAIST, Daejeon, Republic of Korea; 7 Center for Study of Emerging and Re-emerging Viruses, Korea Virus Research Institute, Institute for Basic Science (IBS), Daejeon, Republic of Korea

**Keywords:** Viral infection, Monocytes and macrophages, SARS-CoV-2

## Abstract

Few studies have used a longitudinal approach to describe the immune response to SARS-CoV-2 infection. Here, we perform single-cell RNA sequencing of bronchoalveolar lavage fluid cells longitudinally obtained from SARS-CoV-2-infected ferrets. Landscape analysis of the lung immune microenvironment shows distinct changes in cell proportions and characteristics compared to uninfected control, at 2 and 5 days post-infection (dpi). Macrophages are classified into 10 distinct subpopulations with transcriptome changes among monocyte-derived infiltrating macrophages and differentiated M1/M2 macrophages, notably at 2 dpi. Moreover, trajectory analysis reveals gene expression changes from monocyte-derived infiltrating macrophages toward M1 or M2 macrophages and identifies a macrophage subpopulation that has rapidly undergone SARS-CoV-2-mediated activation of inflammatory responses. Finally, we find that M1 or M2 macrophages show distinct patterns of gene modules downregulated by immune-modulatory drugs. Overall, these results elucidate fundamental aspects of the immune response dynamics provoked by SARS-CoV-2 infection.

## Abstract (web summary)

A longitudinal analysis of SARS-CoV-2 infection in humans is challenging. Here the authors show a single cell RNA-sequencing analysis of BAL fluid cells from ferrets and characterise the time dependent recruitment of macrophage subsets to the lungs in response to SARS-CoV-2 infection.

## Introduction

During the current coronavirus disease-19 (COVID-19) pandemic^1^, cross-sectional research has rapidly broadened our understanding of the immune response to severe acute respiratory syndrome coronavirus 2 (SARS-CoV-2). Immune landscape studies have revealed the pathogenesis of severe COVID-19 with a hyper-inflammatory response^2–4^, and the innate, humoral, and T-cell response of COVID-19 patients have been extensively characterized^5–7^. Currently, ongoing studies are examining the mechanisms of therapeutic modalities, including anti-viral and anti-inflammatory agents, with accompanying clinical trials^8–11^. However, due to the intrinsic limitations of observational studies of human subjects, it is rare to obtain a longitudinal description of the immune response from the initial stage to the resolution of SARS-CoV-2 infection.

In recent studies, single-cell RNA sequencing (scRNA-seq) of bronchoalveolar lavage (BAL) fluid from patients with COVID-19 has provided valuable information on the microenvironment of immune responses to SARS-CoV-2^12–14^. Intriguingly, increased levels of a macrophage subtype originated from circulating monocytes were observed during the inflammatory phase of COVID-19^12^. In addition, we recently demonstrated that peripheral monocytes from severe COVID-19 patients were highly activated, showing strong interferon-mediated inflammatory responses^4^. These findings suggest that both monocytes and macrophages are major cell populations of interest in COVID-19 pathogenesis and patients’ anti-viral response. However, most currently available transcriptomic analyses of immune cells are from cross-sectional studies and, importantly, cannot compare infected status with uninfected status due to the lack of data obtained prior to the SARS-CoV-2 infection. Moreover, BAL invasiveness hinders the acquisition of sequential specimens from critical patients during SARS-CoV-2 infection. These limitations can be overcome by analyzing animal models for the infection with SARS-CoV-2.

The ferret (*Mustela putorius* furo) is widely used as an animal model for investigations of respiratory virus pathogenesis^15,16^. Since ferrets’ natural susceptibility to the influenza virus was discovered in 1933, these animals have been used to recapitulate the course of several human respiratory viral diseases, including parainfluenza virus, respiratory syncytial virus, and SARS-CoV^17^. Moreover, their histoanatomical features—including the ratio between the upper and lower respiratory tract lengths, airway glandular density, and terminal bronchiole structure—provide optimal conditions for mimicking human respiratory infection^17^. We recently reported that a ferret model can reproduce a common natural course of COVID-19 in humans, showing effective infection and rapid transmission^18^. SARS-CoV-2-infected ferrets initially exhibit body temperature elevation and weight loss with viral shedding. In addition, peak viral titer is observed during 2–4 days post-infection (dpi), and after then, the resolution phase which is characterized by body temperature normalization and decrease of viral titer is continued up to 10 days.

Here, we perform scRNA-seq of sequential BAL fluid samples, which is useful for investigating the immunological changes in the lung, from SARS-CoV-2-infected ferrets, compared to the negative control, at 2 days post-infection (dpi) (early stage of SARS-CoV-2 infection with peak viral titer), and 5 dpi (resolution phase with histopathology). Landscape analysis of the ferret lung immune microenvironment reveals dynamic changes in the proportions and characteristics of immune cells over this time. Specifically, we delineate the macrophage population into ten distinct subpopulations based on unique gene expression patterns and describe their chronological transcriptome changes. Intriguingly, rather than tissue-resident alveolar macrophage populations, we find that infiltrating macrophages differentiate into M1 or M2 macrophages after SARS-CoV-2 infection. Moreover, the different spectrums of M1 or M2 macrophages show distinct patterns of gene modules down-regulated by immune-modulatory drugs.

## Results

### Single-cell transcriptomes of BAL fluid cells from SARS-CoV-2-infected ferrets

**Fig. 1:**
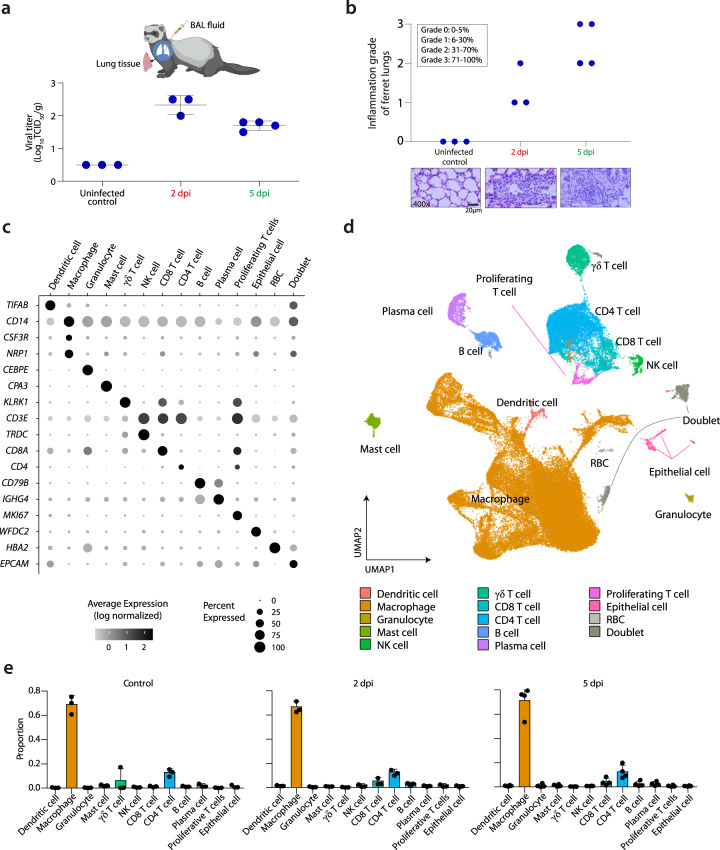
Single-cell transcriptomes of BAL fluid cells from SARS-CoV-2-infected ferrets. **a** Summary of experimental conditions with viral titers in the negative control, at 2 days post-infection (dpi) and 5 dpi. **b** Histopathologic scoring of the lung tissues of negative control ferrets, and SARS-CoV-2-infected ferrets on 2 and 5 dpi. The scale bar indicates 20 μm. **c** Fourteen different clusters and their specific marker gene expression levels, where brightness indicates log-normalized average expression, and circle size indicates percent expressed. **d** UMAP of 59,138 cells from the bronchoalveolar lavage (BAL) fluid of 10 ferrets, colored to show annotated cell types. **e** The proportion of each cell type at uninfected control (*n* = 3), 2 dpi (*n* = 3), and 5 dpi (*n* = 4). NK natural killer, RBC red blood cell, TCID_50_ median tissue culture infectious dose. The height of bars indicates mean and error bars indicate standard deviation. Source data are provided as a Source Data file.

Ferrets were inoculated intranasally with SARS-CoV-2, using a previously described strain isolated from a COVID-19 patient in South Korea^18^. BAL fluid cells and contralateral lung tissue samples were collected by sacrificing infected ferrets at three different time-points: before SARS-CoV-2 infection (uninfected control, *n* = 3), 2 dpi (*n* = 3), and 5 dpi (*n* = 4) (Fig. 1a).

Histopathological analysis and viral shedding clearly indicated SARS-CoV-2 infection (Fig. 1a, b). The infectious viruses were detected in lung tissue at 2 dpi (mean 2.3 log_10_ TCID_50_/g) and 5 dpi (mean 1.6 log_10_ TCID_50_/g). Viral RNA copy number in the BAL fluid also peaked at 2 dpi (Supplementary Fig. 1). Histopathological examinations revealed a pattern of acute pneumonia, characterized by more prominent immune cell infiltration in the alveolar wall and bronchial epithelium at 5 dpi compared to at 2 dpi or in controls, which is consistent with our recent study^18^. Bronchitis was also found near the intact bronchial lining cells at 2 dpi (red circle, Supplementary Fig. 2). Macrophages and neutrophils were the most common infiltrated immune cells, as determined by their typical morphological phenotypes. Semi-quantitative grading confirmed these changes in all the individual histological findings from the ferret subjects (Fig. 1b). Therefore, we categorized the 2 dpi specimens as the early stage of SARS-CoV-2 infection with peak viral titer, while 5 dpi specimens may represent as resolution phase with decreasing viral titer and evident histopathological changes.

Using the 10× Genomics platform, we performed scRNA-seq of BAL fluid cells from 10 ferrets, analyzing a total of 59,138 cells after filtering dead cells. We detected a mean of 8760 UMIs and an average of 2158 genes per cell. By analyzing 59,138 cells with a uniform manifold approximation and projection (UMAP) algorithm based on variable genes with the Seurat package^19^, we identified 28 different clusters (Supplementary Fig. 3a), which were assigned to 14 different cell types expressing representative marker genes (Fig. 1c, Supplementary Fig. 3b–d, Supplementary Data 1). We excluded two clusters with doublet and red blood cells, and thus focused on the following 12 clusters for downstream analysis: dendritic cells, macrophages, granulocytes, mast cells, natural killer (NK) cells, γδ-T cells, CD8^+^ T cells, CD4^+^ T cells, proliferating T cells, B cells, plasma cells, and epithelial cells (Fig. 1d). These clusters and annotated cell types were unbiased according to experimental batches of scRNA-seq (Supplementary Fig. 3e). Although the SARS-CoV-2 RNA sequence was rarely detected, they were contained by the macrophage and epithelial cell clusters (Supplementary Fig. 3f).

To analyze the time-course and dynamic changes of immune responses to SARS-CoV-2, we compared the relative proportions of each cell type in control, 2 and 5 dpi. Analyzing the pattern of proportion changes revealed that the macrophage population comprised the majority of BAL fluid cells over 60% (Fig. 1e). The pattern of each cell type proportion was not evidently changed regardless of time point (Fig. 1e).

### Quantitative and qualitative changes in NK cells and CD8^+^ T cells

**Fig. 2:**
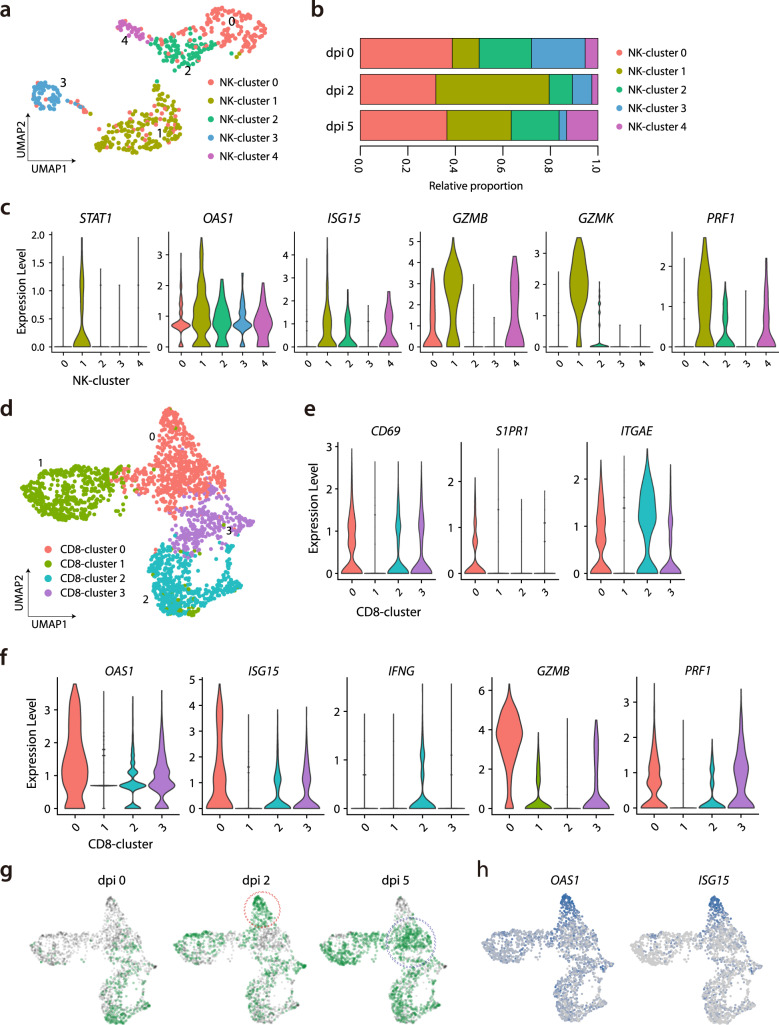
Subpopulation analysis of NK cells and CD8^+^ T cells. **a** UMAP plot of the NK cell subpopulations in all groups, colored to indicate cluster information. **b** Proportion of each cell type in NK cell clusters at uninfected control (*n* = 3), 2 dpi (*n* = 3), and 5 dpi (*n* = 4). **c** Violin plots showing expression levels of *STAT1*, *OAS1*, *ISG15*, *GZMB*, *GZMK*, and *PRF1* in the five NK cell clusters. **d** UMAP plot of the CD8^+^ T-cell subpopulations in all groups, colored to show cluster information. **e**, **f** Violin plots showing expression levels of *CD69*, *S1PR1*, *ITGAE*, *OAS1*, *ISG15*, *IFNG*, *GZMB*, and *PRF1* in the four CD8^+^ T cell clusters. **g** UMAP plot in which color density reflects the distributions of CD8^+^ T cells ferrets in the negative control, at 2 dpi and 5 dpi with SARS-CoV-2. The red circle indicates a concentrated area of cluster 0 with CD8^+^ T cells at 2 dpi, and the blue circle indicates that of CD8^+^ T cells at 5 dpi. **h** UMAP plots show normalized expressions of *OAS1* and *ISG15* in CD8^+^ T cells. Source data are provided as a Source Data file.

As we aimed to investigate immunological changes during the early stage of SARS-CoV-2 infection, we first analyzed NK cells, the representative innate cytotoxic lymphocytes in anti-viral response. Among NK cells, five subclusters were identified from UMAP (Fig. 2a; Supplementary Data 2). With regards to the proportions of each NK cluster, NK cluster 0 was decreased after SARS-CoV-2 infection, NK cluster 1 was increased at 2 dpi but decreased at 5 dpi, and NK clusters 2 and 3 were reciprocally changed (Fig. 2b, Supplementary Fig. 4a). To characterize the activated status of each NK cluster, we performed gene set enrichment analysis (GSEA) using interferon (IFN)-α or IFN-γ responsive signatures. NK clusters 0 and 1 featured prominent responses to interferon IFN-α or IFN-γ (Supplementary Fig. 4b). Indeed, NK cluster 1 showed predominant expression of IFN-stimulated genes including *STAT1*, *OAS1*, and *ISG15* (Fig. 2c). In addition, genes of cytotoxic molecules including *GZMB*, *GZMK*, and *PRF1* were also highly expressed (Fig. 2c)—indicating that NK cluster 1 was IFN-stimulated and activated NK cells. Collectively, the NK cell cluster exhibited activated subclusters with IFN-stimulated and cytotoxic features, which underwent longitudinal changes peaked at 2 dpi.

In addition, we analyzed CD8^+^ T cells, another cytotoxic lymphocyte population, and identified four subclusters from UMAP (Fig. 2d; Supplementary Data 3). The proportion of CD8^+^ cluster 2 tended to decrease at 2 dpi and to increase at 5 dpi, while the proportion of CD8^+^ cluster 0 reciprocally changed (Supplementary Fig. 4c). When we characterize each CD8^+^ cluster, CD8^+^ clusters 2 and 3 exhibited higher expression levels of *CD69* and *ITGAE*, and lower expression of *S1PR1*, reflecting tissue-resident features (Fig. 2e). CD8^+^ cluster 2 showed higher expressions of *CD69* and *ITGAE*, as well as high expression of *IFNG*. These findings were consistent with human CD8^+^ resident memory T (T_RM_) cells, which rapidly induce IFN-γ production using preformed mRNA^20^. Similar to NK cluster 1, CD8^+^ cluster 0 exhibited prominent expression of IFN-stimulated genes (including *OAS1* and *ISG15*) and the genes of cytotoxic molecules (including *GZMB* and *PRF1*) (Fig. 2f). These findings indicated that CD8^+^ cluster 0 comprised activated CD8^+^ cells; however, these cells expressed scarce amounts of *IFNG*. CD8^+^ cluster 0 showed different distributions at 2 dpi (red circle) and 5 dpi (blue circle) (Fig. 2g), which was reflected by higher IFN-stimulated signatures, including *OAS1* and *ISG15* at 2 dpi (Fig. 2h). Considering that antigen-specific T cell response requires a few weeks to take place after infection, those activation patterns at 2 dpi could be led by bystander activation of tissue-resident T cells.

### Sequential changes in macrophage populations during SARS-CoV-2 infection

**Fig. 3:**
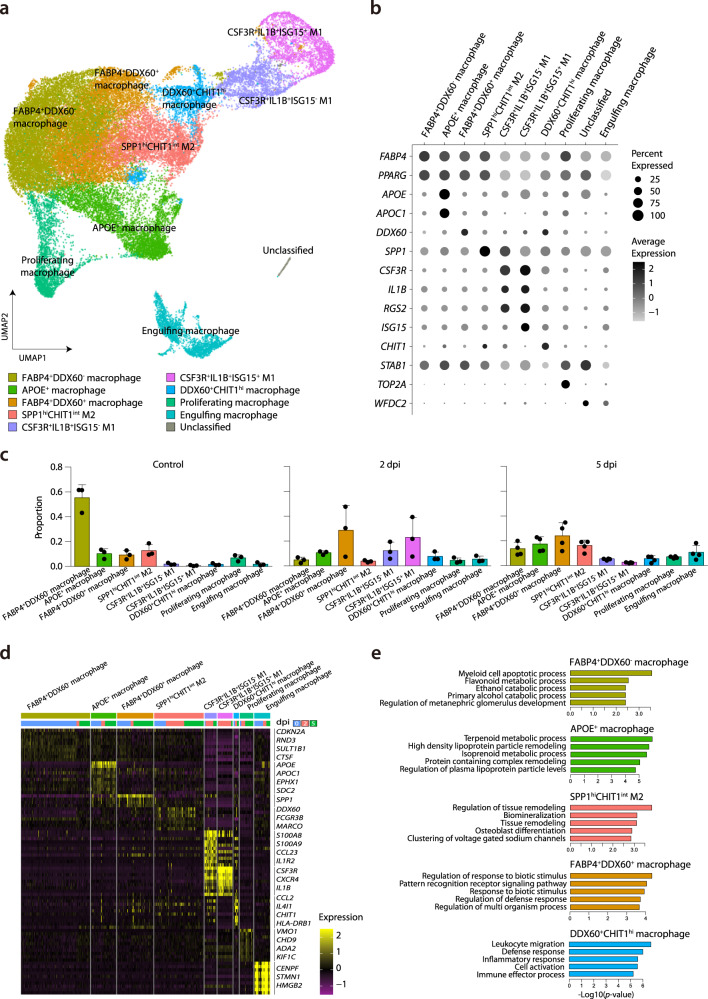
Sub-clustering analysis of macrophages. **a** UMAP plot of the macrophage subpopulations in all groups, colored to show cluster information. **b** Ten different clusters and their specific marker gene expression levels, with brightness indicating log-normalized average expression, and circle size indicating the percent expressed. **c** Proportion of each macrophage cell type at uninfected control (*n* = 3), 2 dpi (*n* = 3), and 5 dpi (*n* = 4). The height of bars indicates mean and error bars indicate standard deviation. **d** Heatmap of cluster-specific differentially expressed genes (DEGs), for each macrophage cell type (*n* = 9). The color indicates the relative gene expression, and representative genes are shown together. **e** Bar plots showing −log10(*p* value) from enrichment analysis of representative GO biological pathways among *FABP4*^+^*DDX60*^−^ macrophages (resting tissue macrophages), *APOE*^+^ macrophages, *SPP1*^hi^*CHIT1*^int^ M2 (potentially profibrogenic), *FABP4*^*+*^*DDX60*^*+*^ macrophages (activated tissue macrophages), and *DDX60*^*+*^*CHIT1*^hi^ macrophages (monocyte-derived infiltrating). The *p* values are calculated from a theoretical null distribution with a two-sided Wilcoxon signed-rank test. Source data are provided as a Source Data file.

We next studied macrophage-specific features that dynamically changed during SARS-CoV-2 infection, since macrophages consistently comprised the majority of cells regardless of time point (Fig. 1e). To this end, we performed a sub-clustering analysis of the macrophage cluster depicted in Fig. 1d. To annotate cell types, we analyzed 40,241 cells using the UMAP algorithm based on variable genes with the Seurat package^19^ and identified 17 different sub-clusters (Supplementary Fig. 5a). Based on signature genes, we selected the following ten macrophage clusters with respective biological significance for downstream analysis: *FABP4*^+^*DDX60*^−^ macrophages (resting tissue macrophages), *APOE*^+^ macrophages, *FABP4*^+^*DDX60*^+^ macrophages (activated tissue macrophages), *SPP1*^hi^*CHIT1*^int^ M2 (potentially profibrogenic), *DDX60*^*+*^*CHIT1*^*hi*^ macrophages (monocyte-derived infiltrating), *CSF3R*^*+*^*IL1B*^*+*^*ISG15*^*−*^ (weakly activated M1), *CSF3R*^*+*^*IL1B*^*+*^*ISG15*^*+*^ (highly activated M1), proliferating macrophages, engulfing macrophages, and unclassified cells (Fig. 3a, b, and Supplementary Fig. 5b). Supplementary Data 4 lists the specific markers used to define each macrophage sub-cluster. Supplementary Fig. 5c displays the normalized expression levels of representative marker genes of each cluster.

The proportion of each lung macrophage subtype underwent distinctive changes. The sub-population of *FABP4*^+^*DDX60*^−^ macrophages (resting tissue macrophages) was dominant in control samples but was drastically decreased at 2 dpi, and partially recovered at 5 dpi (Fig. 3c, Supplementary Fig. 5b). At 2 dpi, we observed increased proportions of *FABP4*^+^*DDX60*^+^ macrophages, *CSF3R*^+^*IL1B*^+^*ISG15*^−^, *CSF3R*^+^*IL1B*^+^*ISG15*^+^, and *DDX60*^+^*CHIT1*^hi^ macrophages. At 5 dpi, the sub-populations of *FABP4*^*+*^*DDX60*^*−*^ macrophages, *APOE*^+^ macrophages, *FABP4*^+^*DDX60*^+^, and *SPP1*^hi^*CHIT1*^int^ M2 constituted major proportions of the samples, while the proportion of M1 macrophages was lower than at 2 dpi. Dynamic changes of the proportions of macrophage subclusters were summarized on UMAP (Supplementary Fig. 5d), and viral-read-containing cells were mainly concentrated in the engulfing macrophage cluster (Supplementary Fig. 5e).

To characterize the subtypes of macrophages in detail, we identified cluster-specific differentially expressed genes (DEGs) (Fig. 3d), and the top 50 DEGs for each cluster were analyzed in terms of gene ontology (GO) biological pathways (Fig. 3e and Supplementary Fig. 5f). DEGs of *FABP4*^+^*DDX60*^−^ macrophages (the dominant population before SARS-CoV-2 infection) were enriched in GO terms, including “myeloid cell apoptotic process” and metabolism-associated pathways (Fig. 3e). *APOE*^+^ macrophages had DEGs that were enriched in GO terms mainly associated with lipoprotein metabolism. As expected, DEGs of *SPP1*^hi^*CHIT1*^int^ M2 macrophages were prominently enriched in GO terms, including “regulation of tissue remodeling” and biological adhesion, indicating that this subtype is associated with the recovery phase of inflammation. In contrast, *FABP4*^+^*DDX60*^+^ and *DDX60*^+^*CHIT1*^hi^ macrophages exhibited DEGs enriched for GO terms associated with activated innate immune response. Supplementary Fig. 5f summarizes the enriched GO terms originated from DEGs of other macrophage sub-clusters. Overall, we defined ten different subtypes of macrophages in SARS-CoV-2 infection, which displayed extensive heterogeneity.

### Transcriptomic changes in each macrophage subpopulation

Since we observed distinctive proportional changes in the lung macrophage subtypes during SARS-CoV-2 infection (Fig. 3c), we next focused on changes in the transcriptome between 2 and 5 dpi in each macrophage subpopulation. *FABP4*^+^*DDX60*^−^ and *FABP4*^+^*DDX60*^+^ macrophages exhibited fewer DEGs than the other macrophage sub-clusters at 2 and 5 dpi (Supplementary Fig. 6a, b). On the other hand, *DDX60*^*+*^*CHIT1*^hi^ macrophages showed remarkably increased numbers of DEGs at both 2 and 5 dpi, and exhibited increased expressions of IFN-responsive genes, such as *OAS1*, *ISG15*, and *RSAD2*, at 2 dpi compared to 5 dpi (Supplementary Fig. 6a). *DDX60*^*+*^*CHIT1*^hi^ macrophages exhibited higher expressions of inflammatory markers or mediators, including *HLA-DRB1*, *MRC1*, and *SERPINE2*, at 5 dpi than at 2 dpi. In differentiated macrophage sub-clusters, including M1 and M2 macrophages, the dynamicity of gene expression change was consistently higher at 2 dpi than 5 dpi (Supplementary Fig. 6b). M1 macrophage clusters showed increased expression of pro-inflammatory genes (including *IL1B*, *CCL8*, and *DUSP1*) and IFN-responsive genes (*OAS1*, *ISG15*, *ISG20*, and *RSAD2*) ﻿ at 2 dpi compared to 5 dpi (Supplementary Fig. 6b). *SPP1*^hi^*CHIT1*^int^ M2 macrophages had different DEGs at 2 dpi, including *SCD*, *CHIT1*, and *IL4I1* (Supplementary Fig. 6b). Therefore, *DDX60*^*+*^*CHIT1*^hi^ macrophages and differentiated M1 and M2 macrophages exhibited increased and distinctive DEG patterns especially at 2 dpi, the peak of viral titer in SARS-CoV-2 infection.

### Different spectrums of M1 or M2 macrophages revealed by RNA dynamics

**Fig. 4:**
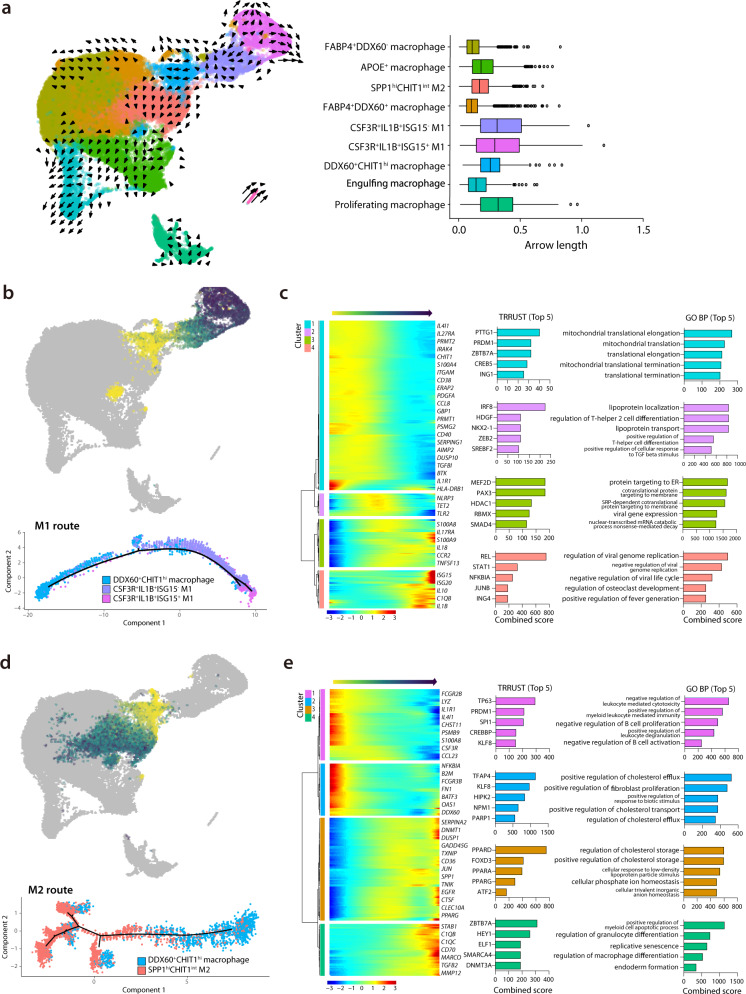
Trajectory analysis from MDIM to M1 and M2 macrophages. **a** Left panel shows a UMAP plot of RNA velocity of macrophage subpopulations. Arrow direction and length indicate qualitative and quantitative changes, respectively. The right panel shows box-plots of ranges (horizontal line), interquartile ranges (boxes), and medians (vertical lines) of arrow length using randomly subsampled cells (1/10 of total cells) included in each cluster in the left panel. **b** Pseudotime trajectory initiated from monocyte-derived infiltrating macrophages (MDIM) toward M1 macrophages (M1 route). **c** Left panel shows relative expression patterns of representative genes in the M1 route plotted along the pseudo time. The color indicates the relative gene expression calculated by Monocle 2. The right panel shows bar plots of the combined scores in the top-five enrichment analysis of the TRRUST database for transcription factor analysis, and representative GO biological pathways in clusters 1–4, as defined in the left panel. **d** Pseudotime trajectory initiated from MDIM toward M2 macrophages (M2 route). **e** Left panel shows relative expression patterns of representative genes in the M2 route plotted along the pseudo time. The right panel shows bar plots of combined scores in the top-five enrichment analysis of the TRRUST database for transcription factor analysis, and the representative GO biological pathways in clusters 1–4, as defined in the left panel. Source data are provided as a Source Data file.

To further evaluate the RNA dynamics of the macrophage cell subpopulations, we analyzed RNA velocity^21^. The RNA velocity approach is an objective and superior method for the analysis of time-resolved phenomena in single-cell transcriptome data using a kinetic model of RNA transcription and splicing. Few kinetics were observed in resting tissue macrophages or activated tissue macrophages, while complex kinetics were formed among monocyte-derived infiltrative macrophages and in both M1 populations (Fig. 4a). To quantify the kinetic dynamics of RNA velocities, we calculated the length of the arrow in Fig. 4a (right panel), which represents the RNA velocities. High-velocity levels were formed in both M1 populations. On the other hand, low levels of dynamics were observed in *FABP4*^*+*^*DDX60*^*+*^ macrophages (activated tissue macrophages), similar to the *FABP4*^*+*^*DDX60*^*−*^ macrophages (resting tissue macrophages), which was consistent with the findings from UMAP embedding (shown in Fig. 4a). Next, we analyzed the direction of the arrow, to investigate the interactions between various clusters. We observed an arrow pointing toward the *SPP1*^hi^ *CHIT1*^int^ M2 cluster from the *DDX60*^*+*^*CHIT1*^hi^ macrophage (monocyte-derived infiltrating) (Fig. 4a), suggesting that the monocyte-derived infiltrating macrophages significantly contributed to the formation of the *SPP1*^hi^*CHIT1*^int^ profibrogenic M2 cluster.

We next investigated the dynamic transcriptome changes from *DDX60*^+^*CHIT1*^hi^ macrophages to M1 or M2 populations. We found that *DDX60*^*+*^*CHIT1*^hi^ macrophages were increased during the acute inflammation period, consistent with a previous study^12^. Using pseudotime analysis for single-cell transcriptomics, we traced the dynamic changes of gene expression from *DDX60*^*+*^*CHIT1*^hi^ macrophages to M1 or M2 macrophages^22^. To validate the trajectory analysis among macrophage subpopulations, we utilized Partition-based graph abstraction (PAGA) mapping analysis, which provides transition graphs estimating connectivity between distinct cell populations based on the single-cell transcriptome^23^. As the PAGA map shows, the *DDX60*^*+*^*CHIT1*^hi^ infiltrating macrophage cluster had two distinct paths to M1 and M2 (Supplementary Fig. 7a). Strong sequential connections between *DDX60*^*+*^*CHIT1*^hi^ infiltrating macrophages and *CSF3R*^*+*^*IL1B*^*+*^*ISG15*^−^ M1 population, and between *CSF3R*^*+*^*IL1B*^*+*^*ISG15*^−^ M1 and *CSF3R*^*+*^*IL1B*^*+*^*ISG15*^*+*^ M1 macrophages were the major routes for the M1 differentiation. These data suggested that separate trajectories to M1 and M2 are useful to describe their distinct pathways from the monocyte-derived infiltrating macrophages.

For the trajectory toward M1 macrophages (M1 route) (Fig. 4b, Supplementary Data 5), we defined four distinctive clusters showing modular gene expression changes. We summarized their top five associated transcription factors using the TRRUST database^24^, and the top five GO biological pathways (GO-BP) (Fig. 4c). Notably, cluster 4 of the M1 route (which was exclusively expressed in highly activated M1 macrophages) showed concurrently increased expressions of *IL1B* and IFN-stimulated genes (*ISG15* and *ISG20*), which were associated with GO terms of enhanced anti-viral activity in the early phase of the immune response. These findings indicated that this gene expression change was part of a natural defense mechanism involving M1 macrophage differentiation (Fig. 4b). The highly activated M1 macrophage cluster showed predominant enrichment of pro-inflammatory mediators, including *IL1B* and *CXCL8* (Supplementary Fig. 7b), which was further supported by our results showing that the highly activated M1 was highly enriched with gene sets from severe COVID-19 patients (Supplementary Fig. 7c). These results suggested that the distinct macrophage subpopulation that was potentially derived from monocyte-derived infiltrating macrophages had rapidly undergone SARS-CoV-2-mediated activation of inflammatory macrophage responses.

For the trajectory toward *SPP1*^hi^*CHIT1*^int^ M2 macrophages (M2 route) (Fig. 4d, Supplementary Data 6), we defined four distinct clusters and analyzed their features with GSEA, as described in Fig. 4e. Cluster 3 of the M2 route showed an increased association with transcription factors of the peroxisome proliferator-activated receptor (PPAR) family (PPAR-δ, PPAR-α, and PPAR-γ) and with pathways associated with cholesterol metabolism. PPAR-γ activation reportedly may drive monocytes toward anti-inflammatory M2 macrophages^25^. Indeed, the next cluster in the pseudo time trajectory, cluster 4 of the M2 route, showed increased expressions of *C1QB*, *C1QC*, *MMP12*, and *TGFB2*, which are known to be key genes of well-differentiated M2 macrophages.

Collectively, the macrophage subpopulations underwent time-dependent and cell-type-specific changes during SARS-CoV-2 infection. These subpopulations exhibited a continuous spectrum of changes, mainly from the monocyte-derived infiltrating macrophages, at the transcriptome level.

### Analysis of specific gene modules originated from M1 and M2 route

**Fig. 5:**
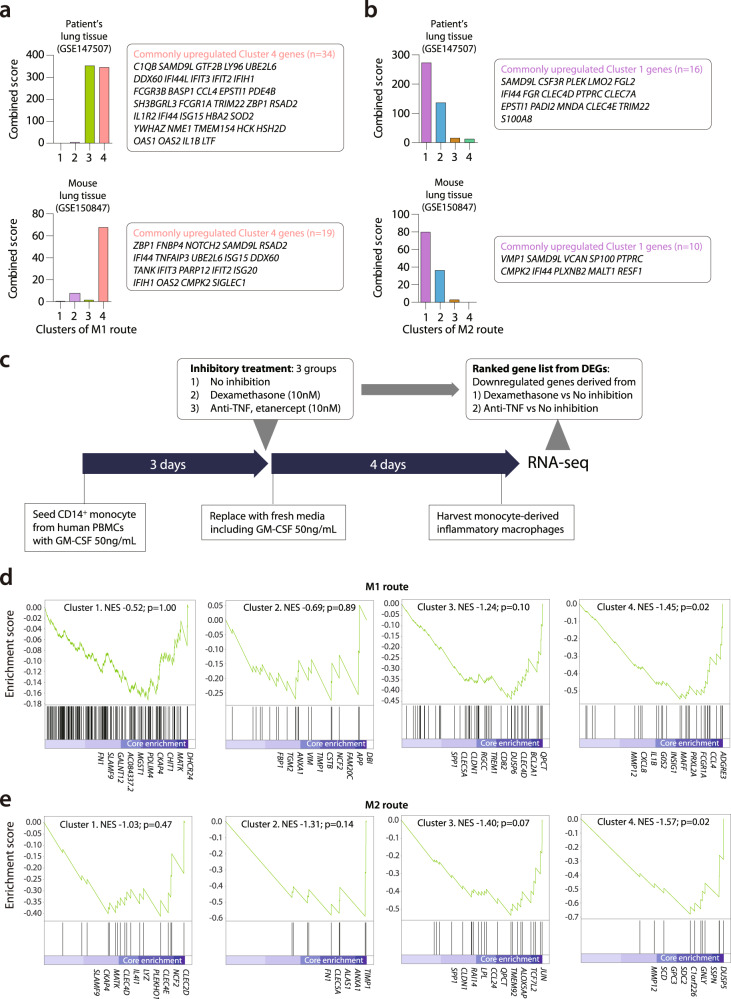
GSEA of gene modules originated from the M1 route and M2 route. **a**, **b** Gene set enrichment analysis (GSEA) of clusters 1–4 of the M1 route a. and M2 route b. using public transcriptome data, including post-mortem lung tissue from a COVID-19 patient (GSE147507) and lung tissue from a SARS-CoV-2-infected mouse (GSE150847). For calculating combined scores, upregulated genes derived from COVID-19 patients’ lung tissue and SARS-CoV-2-infected mouse lung were compared to the marker genes of pseudo time clusters of M1 or M2 route, which was calculated from the *p* value obtained using Fisher’s exact test and the *z*-score (see “Methods”). Commonly upregulated genes are listed in the box right side of each bar graph. **c** Experimental design to make dexamethasone and etanercept responsive gene sets for GSEA of clusters 1–4 of the M1 and M2 route. **d**, **e** GSEA of clusters 1–4 of the M1 route (**c**) and M2 route (**d**) using ranked gene list originated from dexamethasone-downregulated DEGs derived from in vitro experiment described in (**c**). The name of genes included as core enrichment was listed, NES normalized enrichment score. The *p* values of the combined score are calculated with a two-sided Fisher’s exact test. The *p* value of GSEA is the probability under the null distribution calculated by the permutation test. Source data are provided as a Source Data file.

Next, we compared the dynamically changed macrophage gene modules from M1 and M2 routes with the previously reported transcriptome changes of COVID-19 patients and SARS-CoV-2-infected experimental models^26,27^. GSEA showed that upregulated gene sets from the postmortem lung tissue of a COVID-19 patient and a SARS-CoV-2-infected mouse were commonly associated with cluster 4 of the M1 route (Fig. 5a). Gene sets from postmortem lung tissue of a COVID-19 patient were also associated with cluster 3 of the M1 route. Among actively differentiating features of the M1 transition (from infiltrating to M1 macrophage), the later phases approaching to M1 population were enriched in the set of upregulated genes from post-mortem lung tissue of COVID-19 patients. In contrast, clusters 1 and 2 of the M2 route were highly associated with those two gene sets (Fig. 5b). Within the M2 differentiation route, genes in the early phase (cluster 1) were prominently enriched within this lung tissue gene set. GSEA plots using DEGs derived from the transcriptome of post-mortem lung tissue of COVID-19 patients also confirmed these patterns (Supplementary Fig. 8a, b). From these results, we infer that the post-mortem lung tissue exhibited well-differentiated and interferon-stimulated M1 features defined by the trajectory analysis of genes from the SARS-CoV-2 infected ferrets, while the inflammation-resolving M2-driving features were less prominent. This finding might be associated with the poor outcomes in COVID-19 patients with a continuing pro-inflammatory response.

Immune-modulatory treatments, including corticosteroids and cytokine-targeted agents, have been considered as a means of regulating hyper-inflammatory responses in COVID-19 patients; however, the exact immunological features of the target cells affected by these treatments is unclear. To evaluate the effect of immune-modulatory drugs on M1 or M2 differentiation and to validate our RNA velocity analysis, we performed enrichment tests on these trajectory-specific modular gene expressions relative to DEGs that represent immunosuppression derived from an in vitro experiment (Fig. 5c). Following an in vitro differentiation protocol using GM-CSF treatment for pro-inflammatory macrophages, we tested how immunosuppression impacts human CD14^+^ monocytes (*n* = 4) by applying three different conditions: no inhibition, dexamethasone, and anti-TNF agent treatment. We then performed RNA-sequencing of the harvested cells and analyzed DEGs obtained from the dexamethasone group and the anti-TNF group compared to those from the group that received no inhibition. Those two sets of DEGs were used for GSEA of four clusters from the M1 route, and other four clusters of the M2 route (Fig. 5d, e; Supplementary Fig. 8c, d). Interestingly, we observed similar trends in both M1 and M2 routes. The gene sets originated from the later phases of M1 route (Cluster 4, normalized enrichment score (NES)—1.45, *p* = 0.02, permutation test) and the M2 route (Cluster 4, NES—1.57, *p* = 0.02, permutation test) were more strongly enriched among the DEGs induced by dexamethasone-mediated suppression (Fig. 5d, e; Supplementary Data 7). GSEA using DEGs induced by anti-TNF treatment revealed a similar tendency, but significant enrichment was only found in clusters 3 and 4 of the M2 route (NES—1.54, *p* = 0.02 and NES—1.49, *p* = 0.03, respectively, permutation test) (Supplementary Fig. 8c, d; Supplementary Data 8). In summary, we found that immunosuppressive treatments, including dexamethasone and anti-TNF, were shown to downregulate the differentiation of macrophages during the later phases of M1-to-M2 transition.

## Discussion

Although recent studies have reported the single-cell transcriptome of BAL fluid cells cross-sectionally obtained from COVID-19 patients, none have used a longitudinal approach along with the natural disease course. Examining BAL fluid cells can provide additional information complementary to examining lung tissue in a few aspects. First, BAL is a sampling technique used to obtain a living immune cell suspension from the lower respiratory tract, which can be readily analyzed using current immunological research tools, including flow cytometry and single-cell sequencing. Studying BAL fluid cells is an efficient way to focus on the immune landscape of viral infection. However, lung tissue also provides more precise information about the degree of immune cell infiltration rather than BAL fluid. Second, analyzing BAL fluid is a more favorable diagnostic approach for human patients than collecting lung tissue samples. BAL is less invasive than surgical biopsy of lung tissue. Additionally, compared to tissue biopsy, BAL fluid may provide more unbiased information regarding the targeted part of lung tissue. Thus, analyzing the immune landscape in the BAL fluid of an animal model could be useful to facilitate the translation of findings from animal studies to human disease pathogenesis.

In the present study, we investigated single-cell transcriptome changes throughout SARS-CoV-2 infection using BAL fluid from a ferret model. We found that specific sub-clusters of NK cells and CD8^+^ T cells exhibited increased responses to IFN, especially at 2 dpi, while their intrinsic cytotoxic properties against viral infection were preserved. However, considering that antigen-specific response requires a few weeks to be initiated after infection, those activation patterns at 2 dpi could be led by bystander activation of tissue-resident T cells. Relatively late time-points (14 dpi or 28 dpi) would be required to analyze the SARS-CoV-2-specific B cell and T cell response. More importantly, among macrophages—the major population of BAL fluid cells—we identified 10 different subpopulations that exhibited relative proportion changes from 0 to 5 dpi. The predominant dynamic changes of the transcriptome involved monocyte-derived infiltrating macrophages and differentiated M1/M2 macrophages, especially at 2 dpi. We also observed distinctive and stepwise differentiation from monocyte-derived infiltrating macrophages toward M1 or M2 macrophages.

Our present results included observation of IFN-responsive signatures, regardless of immune cell type, mostly at 2 dpi. The presence of an IFN-responsive signature has also been reported in previous transcriptome studies of SARS-CoV-2 infection^3,4,12^. Data are controversial regarding the relationship between IFN response strength and COVID-19 severity—delayed but robust expression of IFN-associated genes might provoke harmful immunopathology, but their early increase is beneficial^28^. Our ferret model mimicked SARS-CoV-2 infection with a clinical course of mild severity and spontaneous recovery. Therefore, our findings suggest that prominently increased expression of IFN-responsive genes at 2 dpi might be beneficial in clearing SARS-CoV-2. This observation is further supported by the observed increase of the IFN-stimulated M1 subpopulation.

The BAL fluid cells from our ferret model comprised a diverse subpopulation of macrophages. We annotated 10 different subpopulations among 17 different clusters based on previous single-cell studies of alveolar macrophages^12,25,29–31^. Based on the RNA velocity analysis, we could infer that the proliferating macrophage cluster was derived from the tissue macrophage clusters, rather than from the monocyte-derived macrophage clusters. This finding is concordant with a previous study that demonstrated maintenance of tissue-resident macrophages regulated by local proliferation in the lung^32^. The presence of the uninfected control group provided an interesting contrast with specific features of activated and differentiated macrophages in later phases. The proportion of *FABP4*^*+*^*DDX60*^*−*^ macrophages (resting tissue macrophages) was near 60% of the macrophage population in control samples and drastically decreased at 2 and 5 dpi, suggesting either that this population underwent a change of transcriptomic features towards another population or the infiltration of a new population from circulation. *FABP4*^*+*^*DDX60*^*−*^ macrophages could have evolved into *FABP4*^+^*DDX60*^+^ activated tissue macrophages; however, the increase of activated tissue macrophages was not sufficient to fully explain the decreased proportion of resting tissue macrophages. Notably, the increased RNA velocity of *DDX60*^+^*CHIT1*^hi^ macrophages (monocyte-derived infiltrating) and M1/M2 macrophages indicated that these were the major populations that underwent dynamic changes after SARS-CoV-2 infection. Here, we found that with regards to the changing macrophage populations, *FABP4*^*+*^*DDX60*^*−*^ macrophages decreased after inoculation but were not restored later, and M2 macrophages were increased and remained a major population from 2 to 5 dpi. These findings indicate that during the viral resolution phase, an active repair process is underway rather than complete recovery to preinfection status.

Immuno-modulatory treatments—including corticosteroids and targeted agents, such as Janus kinase inhibitors—have been considered to regulate hyper-inflammatory responses in COVID-19 patients^9,10,28^. To apply such treatments in heterogeneous COVID-19 patients, we must understand the exact features and proportions of the target immune cell populations that will be affected. Along the transcriptome continuum of monocyte-derived infiltrating *DDX60*^*+*^*CHIT1*^hi^ macrophages to M1 macrophages (the M1 route) or M2 macrophages (the M2 route), we found that the later clusters, similar to more differentiated macrophages, were enriched in the ranked DEGs downregulated by corticosteroid. Corticosteroid therapy reduces mortality in cases of severe pneumonia^33^, and the beneficial role of dexamethasone in hospitalized COVID-19 patients has also been reported recently^34^. Our current findings elucidate the exact macrophage subpopulations affected by these immuno-modulatory treatments.

Overall, our present study provides fundamental information regarding the immune response dynamics provoked by SARS-CoV-2 infection, as well as a detailed description of the natural course and changes of macrophages in the ferret model.

## Methods

### Experimental ferrets

Experiments were performed using 14- to 20-month-old female ferrets (*n* = 10, ID Bio Corporation, Cheongju, Korea) that were serologically negative for influenza A viruses (H1N1 and H2N2), MERS-CoV, and SARS-CoV. Ferrets were maintained in the isolator (Woori IB Corporation, Daejeon, Korea) in BSL3 of Chungbuk National University. All ferrets were group-housed with a 12-h light/dark cycle and allowed access to food and water. After two days of adaption to BSL3 conditions, the ferrets were intranasally inoculated with phosphate-buffered saline (PBS) (*n* = 3) or 10^5.8^ TCID_50_/mL of NMC-nCoV02 (*n* = 7), while under anesthesia with ketamine (20 mg/kg) and xylazine (1.0 mg/kg). All animal studies were conducted following protocols approved by the Institutional Animal Care and Use Committee (IACUC) of Chungbuk National University (Approval number CBNUA-1352-20-02).

### Virus and cells

SARS-CoV-2 strain NMC-nCoV02^18^ was propagated in Vero cells in Dulbecco’s Modified Eagle Medium (DMEM; GIBCO, NY, US, 11-995-040) supplemented with 1% penicillin/streptomycin (GIBCO, 15140-122) and TPCK-treated trypsin (0.5 μg/mL; Worthington Biochemical, NJ, US, TRSEQZ) in a 37 °C incubator with 5% CO_2_ for 72 h. The propagated virus was then stored at −80 °C, and used as the working stock for animal studies. The 50% tissue culture infective dose (TCID_50_) was determined via fixation and crystal violet staining.

### Harvesting BAL fluid cells

At 2 and 5 dpi, respectively, three and four ferrets were euthanized, and BAL fluid was collected. As a control group, the three PBS-treated ferrets were euthanized at 2 dpi and BAL fluid was collected. Briefly, with the ferret positioned in dorsal recumbency, 30 mL of cold sterile PBS solution containing 5% fetal bovine serum was injected through the tracheal route and then collected. This collected BAL fluid was centrifuged at 400 × *g* for 10 min at 4 °C. Then the supernatant was removed, and the cell pellet was suspended in 5 mL 10× RBC lysis buffer (Thermofisher, Waltham, MA, USA, 00-4300-54) diluted 1:10 with distilled water, followed by a 10-min incubation at room temperature. After the RBC lysis reaction, 20 mL of 1× PBS was added to stop the lysis reaction, followed immediately by centrifugation at 500 × *g* for 5 min at 4 °C. Then the supernatant was removed, followed by cell number and viability analyses.

### Virus isolation from the lungs of infected ferrets

The virus titers in collected lung tissues were determined by TCID_50_ in Vero cells. Briefly, lung tissue samples were homogenized in an equal volume (1 g/mL) of cold 1× PBS containing 1% penicillin/streptomycin. Tissue homogenates were centrifuged at 5000 × *g* for 15 min at 4 °C, and then the supernatants were serially diluted (10^−^^1^ to 10^−8^) in DMEM. Dilutions of each sample were added to Vero cells, followed by a 2-h incubation. Next, the media (DMEM) was changed, and the cytopathic effects (CPEs) were monitored for 4 days. We determined the TCID_50_ through fixation and crystal violet staining.

### Histology

Lung tissue samples were collected at 2 and 5 dpi, incubated in 10% neutral-buffered formalin for fixation and then embedded in paraffin following standard procedures. The embedded tissues were sectioned and dried for 3 days at room temperature. Then the tissue sections were placed on glass slides, stained with hematoxylin and eosin (H&E), and compared with the PBS control group. Slides were viewed using an Olympus IX 71 (Olympus, Tokyo, Japan) microscope, and images were captured using DP controller software.

### scRNA-seq analysis and basic quality control

Reference sequence and gene information were downloaded from the Ensembl database (MusPutFur1.0, under accession number GCF_000215625.1), and then annotated with human ortholog genes using the same database (Biomart database, GRCh38). The SARS-CoV-2 sequence was downloaded from NCBI GenBank (Wuhan-Hu-1, a widely used reference sequence, under accession number NC_045512). Reference genome information was pre-processed for single-cell data processing using mkref (Cell Ranger; 10× genomics, Pleasanton, CA, US, v3.0.2), and the fastq files were generated through the process of demultiplexing the sequenced data (Cell Ranger). Next, the reads were aligned to the ferret–virus combined reference genome, and the aligned read data were analyzed using Seurat R package v3.1.5^35^. Based on the characteristics of inflammatory tissue and the assumption that viral transcripts can present in dying cells, we did not exclude low-quality cells from the analysis. Ambient RNAs were examined and adjusted using SoupX (10.1101/303727), and were present in 1–3% of each sample, indicating that the samples were relatively clean/washed. We also excluded doublets perceived based on the dual expression of cell-type-specific gene expression markers, which were dominant in the cluster “Doublet.” Despite high variability in the number of UMIs detected per cell, most cells were enriched with UMIs within a reasonable range (interquartile range: 2455–12,764).

In each cell, gene expression was normalized and scaled using the SCTransform algorithm^36^. Dimensional reduction and visualization were performed via principal components analysis (PCA) and UMAP—using the top 20 PCs for whole-cell types, 5 PCs for NK and CD8 T cells, and 13 PCs for monocyte/macrophage cell types—with parameters of min.dist = 0.2, and n.neighbor = 20. Lastly, the cells were clustered by unsupervised clustering, using the default pipeline of the Seurat package (resolution = 0.4 for whole-cell types, 0.3 for NK cells, 0.2 for CD8^+^ T lymphocytes, and 0.6 for monocytes/macrophages). We observed two polymorphic genes that significantly affected the clustering of a subset of macrophages by samples: *HLA-DQA1* and ENSMPUG00000007244, the latter of which is putative *HLA-DQB1* or *HLA-DQB2*, and has a DNA sequence that overlaps 78.03–78.81% with human *HLA-DQB1* or *HLA-DQB2*. We removed these two genes from the count matrix and re-processed, and found that the batch effect was resolved.

### Marker detection and differential expression analysis

To identify marker genes, we selected genes in each cluster that were upregulated relative to the other clusters, based on the Wilcoxon rank-sum test in Seurat’s implementation (FindAllMarkers function), with a >0.25 log fold change compared with the other clusters and a Bonferroni-adjusted *p* value of <0.05. To investigate the dynamic changes in gene expression in certain cell clusters, we tested DEGs, using the Wilcoxon rank-sum test (Supplementary Fig. 6). Gene names that had a human ortholog were marked when the *p* value was <0.05, and the absolute value of the log2 fold change was >0.4.

### GO and pathway enrichment analyses

As shown in Fig. 3e and Supplementary Fig. 5f, cluster-specific expression markers were subjected to GO enrichment analysis^37^, which is based on the performance of Fisher’s exact test on curated gene sets annotated according to the GO consortium in the biological process category. Ontology terms associated with T cells and eosinophils, and near-duplicated terms, were removed using a custom script, with the following exclusion criteria: GO terms, including “T_HELPER|T_CELL”, “EOSINOPHIL”, “POSITIVE”, or “NEGATIVE”. For each cluster, the top 50 genes (prioritized by fold change when comparing each cluster with the rest) were subjected to the enrichment test. Genes that were expressed in >80% of cells in the rest of the clusters were excluded.

To predict transcription factors that might drive macrophage differentiation in pathology, the same enrichment test was performed using the TRRUST transcription factor-target gene database^24^.

### RNA velocity

To investigate the characteristics of RNA dynamics among macrophages in the ferret model, we analyzed RNA velocity based on modeling gene expression induction and repression using spliced and unspliced reads. This technique was previously demonstrated to be feasible in a 3ʹ captured single-cell RNA sequencing library using the velocyto tool^21^. Spliced and unspliced reads were counted using the run10× command in the velocyto tool with default options. The count matrixes were filtered using velocyto’s standard pipeline, with min.max.cluster.average parameters of 0.08 for the spliced read count matrix, and 0.06 for the unspliced read count matrix. Totally, 5000 cells (approximately 1/10 of total cells) were randomly selected among a macrophage/monocyte population of 40,241, 5000 cells with pooling of the 20 nearest neighbors in the spliced/unspliced count matrix. Through this process, the cell distance matrix was derived from Seurat’s shared neighborhood network matrix with default parameters (FindNeighbors function). Velocity estimation was conducted using the options of delta*T* = 1, fit.quantile = 0.05, and kCells = 1 (as k-nearest neighbor pooling was already performed before the random sampling of 5000 cells).

### Analysis of dynamic transcriptome changes accompanying M1 and M2 differentiation

To investigate the dynamic changes along the M1 and M2 differentiation pathways, we exported related cell clusters for monocle’s standard analysis process. The related clusters included weakly activated M1, highly activated M1, and monocyte-derived infiltrating macrophages for the M1 pathway; and monocyte-derived infiltrating macrophages and SPP1^high^CHIT1^int^ profibrogenic M2 for the M2 pathway. Briefly, CellDataSet objects were built based on normalized count (SCTransform), and then processed using estimateSizeFactor and estimateDispersions function (default option), detectGenes (with the min_expr = 0.1 option), setOrderingFilter and reduceDimension (with options of max_components = 3, and method = “DDRTree”), orderCells (default option), and plot_cell_trajectory (default option). Trajectory-specific genes were grouped into four clusters using hierarchical clustering. Finally, each cluster was subjected to further enrichment analysis for transcription regulation or ontology-based analysis.

### RNA-sequencing of cultured cells

Fifty milliliters of peripheral blood was obtained from four human healthy volunteers, who were provided written informed consent. This study was approved by the institutional review board of KAIST (IRB No. KH2018-118). Peripheral blood mononuclear cells (PBMCs) were isolated from whole blood via standard Ficoll-Paque (GE Healthcare, Uppsala, Sweden, 17-5442-02) density gradient centrifugation. Classical monocytes were isolated from PBMCs using the Classical Monocyte Isolation Kit, human (Miltenyi Biotec, Bergisch Gladbach, Germany, 130-117-337) according to the manufacturer’s protocol. Isolated classical monocytes were cultured in 12-well plates with 50 ng/mL of GM-CSF (Peprotech, NJ, US, 215-GM) added to 1.5 mL RPMI medium per well (750,000 cells per well). On day 3, half of the medium from each well of cell culture was replaced with a fresh medium including the immunosuppressive medications (negative control, dexamethasone 10 nM, or Etanercept 10 nM). On day 7, we harvested and cryopreserved the cultured cells in Trizol reagent (Invitrogen, CA, USA, 15596026) and RNA extraction was performed following the manufacturer’s instructions. The total RNA quality was measured by the Agilent 2100 Bioanalyzer System (Agilent, CA, US). Extracted RNA samples were processed using the TruSeq Stranded mRNA Library Prep Kit (Illumina, CA, US, 20020594) and sequenced on an Illumina NovaSeq 6000 (Illumina). Read counts and RNA-Seq by Expectation-Maximization (RSEM)^38^ were calculated and DEGs were analyzed using DESeq2^39^.

### Gene set enrichment analysis

Gene set enrichment analysis (GSEA) was performed using enrichR^27^, which provided a “combined score” as the degree of enrichment. The combined score (*c*) was calculated from the *p* value (*p*) obtained using Fisher’s exact test and the *z*-score (*z*) of the deviation from the expected rank, as in Eq. (1):^40^

1 $$c=\,\log (p)\cdot z$$

For the ranked list of genes, we also utilized GSEA plots with the NES and the *p* value^41^.

### Statistical analysis

The statistical significance of the combined scores from GSEA results was assessed by paired *t* test. Data plotting, interpolation, and statistical analysis were performed using GraphPad Prism 8.2 (GraphPad Software, La Jolla, CA). Statistical details of experiments are described in the Figure legends. A *p* value less than 0.05 is considered statistically significant.

### Reporting summary

Further information on research design is available in the Nature Research Reporting Summary linked to this article.

## Supplementary information

Supplementary Information

Description of Additional Supplementary Files

Supplementary Data 1

Supplementary Data 2

Supplementary Data 3

Supplementary Data 4

Supplementary Data 5

Supplementary Data 6

Supplementary Data 7

Supplementary Data 8

Reporting Summary

## Data Availability

The single cell RNA-sequencing data has been deposited in the GEO database under the primary accession code GSE171828. Sequencing data referred to in this paper are available in the GEO database with the primary accession codes GSE147507 and GSE149689. The data that support the findings of this study are available from the corresponding author upon reasonable request. Source data are provided with this paper.

## Source data

Source Data

## Code availability

All the custom codes used in this study are available at Github and Zenodo (https://github.com/kijong-yi/SARS-CoV-2_Ferret_BAL)^42^.
