# Supplementary material for: Single-cell transcriptome of bronchoalveolar lavage fluid reveals sequential change of macrophages during SARS-CoV-2 infection in ferrets
Source: Nat Commun. 2021 Jul 27;12:4567. doi: 10.1038/s41467-021-24807-0 (PMC8316405; doi:10.1038/s41467-021-24807-0)
Supplement: Supplementary file 1 — Supplementary Information [file 41467_2021_24807_MOESM1_ESM.pdf]

Supplementary Information

Single-cell Transcriptome of Bronchoalveolar Lavage Fluid Reveals Dynamic Change of Macrophages During SARS-CoV-2 Infection in Ferrets

Jeong Seok Lee, June-Young Koh, Kijong Yi, Young-Il Kim, Su-Jin Park, Eun-Ha Kim, Se-Mi Kim, Sung Ho Park, Young Seok Ju, Young Ki Choi, and Su-Hyung Park

Contents:

Supplementary Figures 1 – 8

Supplementary Fig. 1

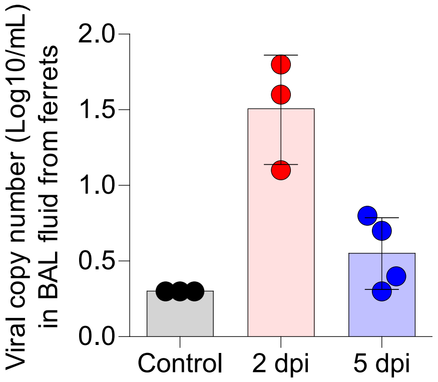

Supplementary Fig. 1. Viral copy number in BAL fluid from ferrets. Viral RNA copy number in bronchoalveolar lavage (BAL) fluids from control ferrets and SARS-CoV-2-infected ferrets on 2 and 5 days post-infection (dpi). Source data are provided as a Source Data file. Each dot indicates each ferret subject. The height of bars indicate mean of viral copy number and error bars indicate their standard deviation.

Supplementary Fig. 2

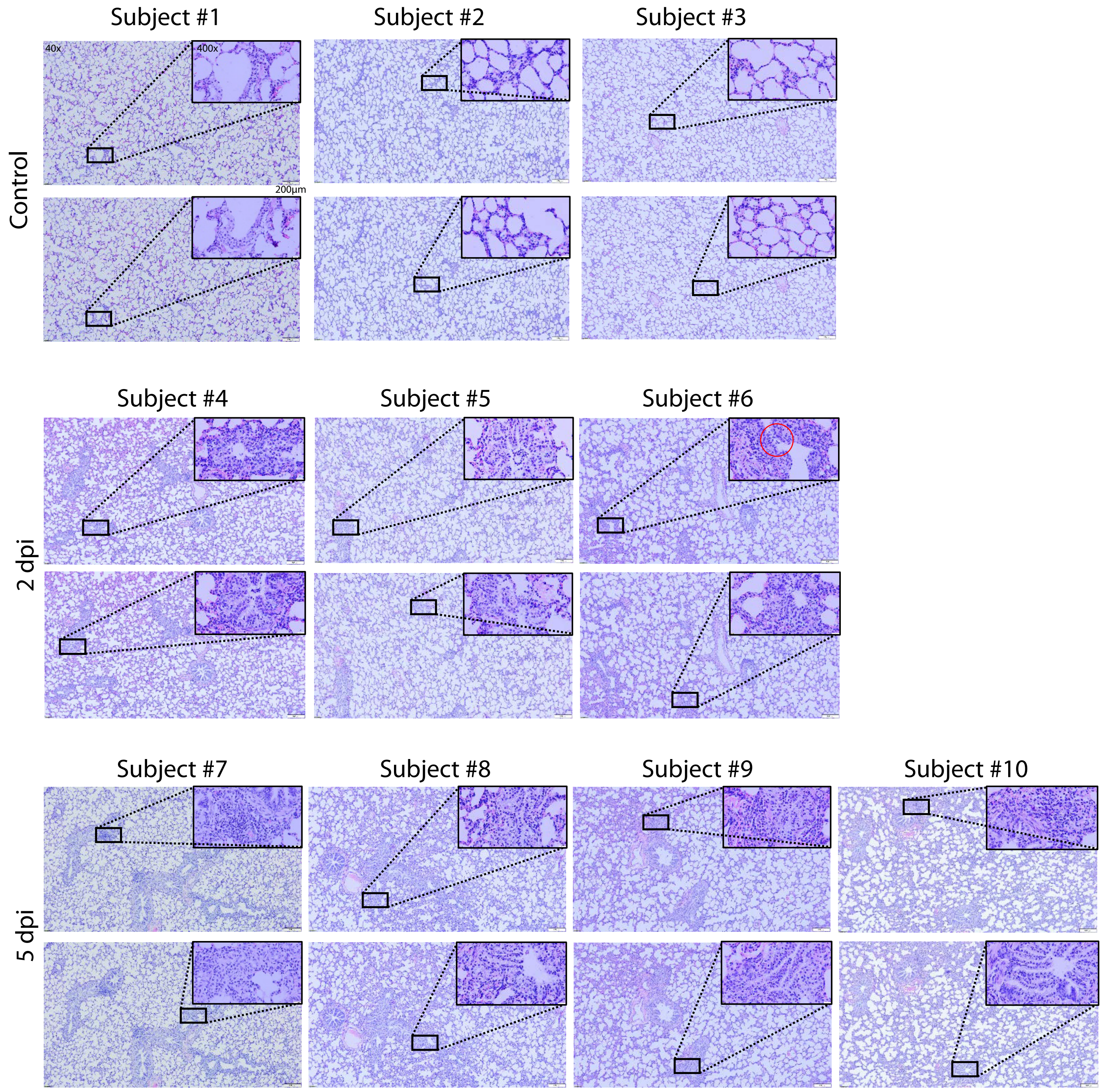

Supplementary Fig. 2. Histologic features of the lung tissues of ferrets. Histologic features of the lung tissues of negative control ferrets (n = 3), and SARS-CoV-2-infected ferrets on 2 (n = 3) and 5 days (n = 4) post-infection (dpi). Prominent bronchitis near the bronchial lining is marked by red circle on the image of subject #6. Two 400x magnified images of each histology slide from all 10 ferret subjects were presented. The scale bars indicate 200μm.

Supplementary Fig. 3

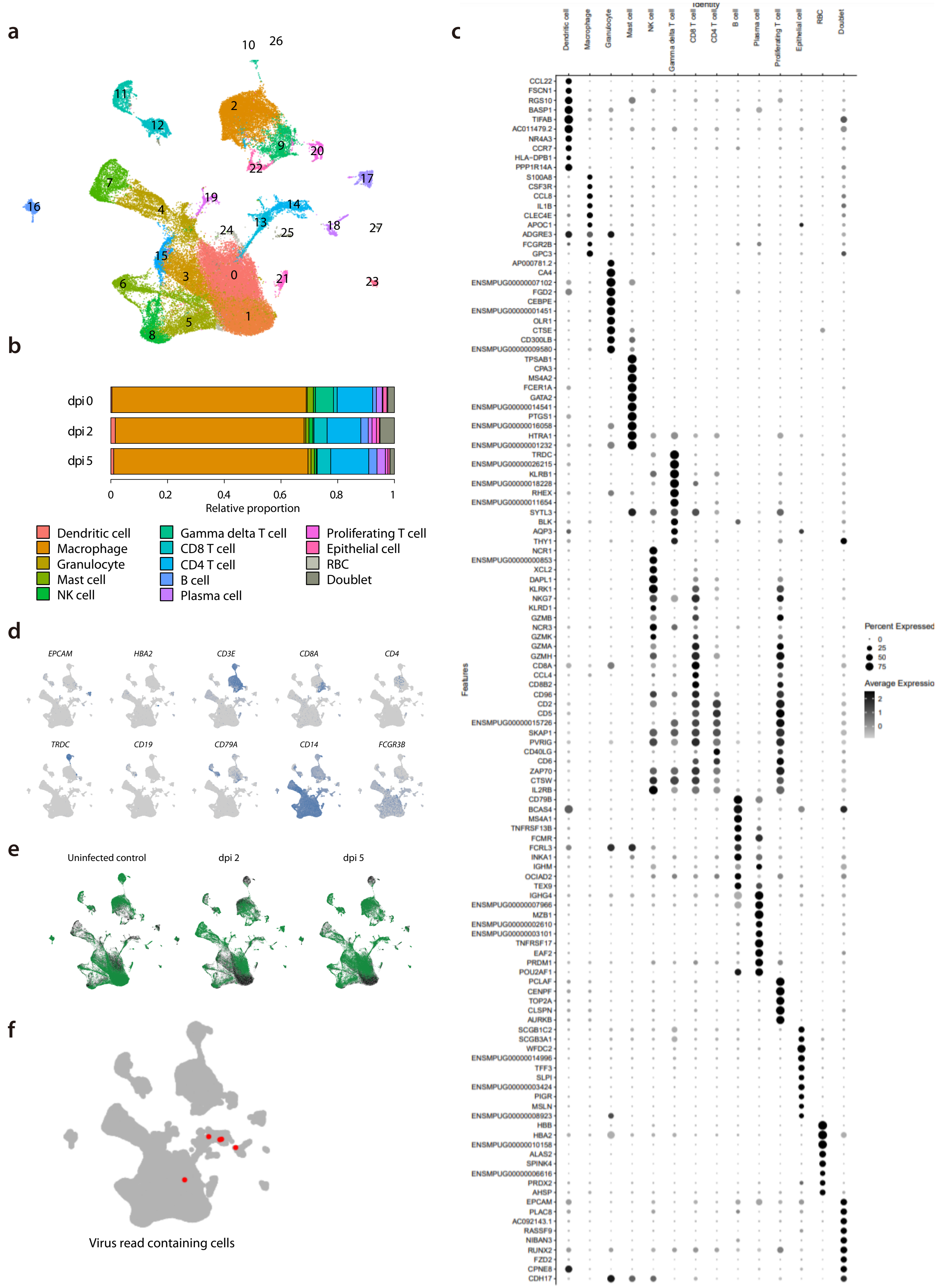

Supplementary Fig. 3 (Cont.)

Supplementary Fig. 3. Clustering of cells obtained from BAL fluid based on single cell transcriptome
a UMAP plot colored according to cluster. **b** Proportion of each cell type at 0 days post-infection (dpi) (n = 3), 2 dpi (n = 3), and 5 dpi (n = 4). **c** Dot blots displaying the top 10 enriched genes of each cluster. **d** UMAP plots showing normalized expression of known markers. **e** UMAP plot, with color density reflecting the distribution of cells at 0, 2, and 5 dpi. **f** UMAP plot of virus-read-containing cells (red dots). Source data are provided as a Source Data file.

Supplementary Fig. 4

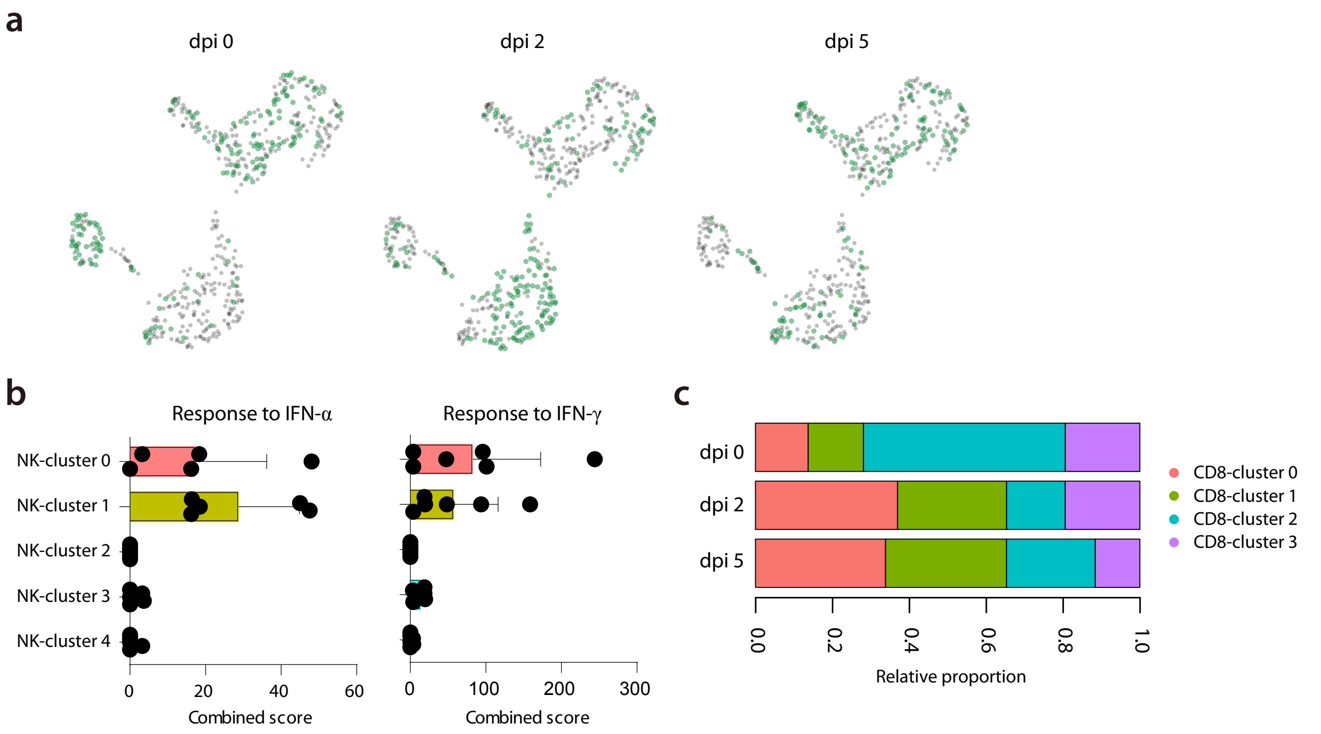

Supplementary Fig. 4. Sub-clustering of CD8^{+} T cells. a UMAP plot, with color density reflecting the distribution of NK cells at 0, 2, and 5 days post-infection (dpi). **b** Bar graphs with mean and standard deviation of 'combined score' showing the results of gene set enrichment analysis on five NK cell clusters using two gene sets: "Response to IFN-α" (left) and "Response to IFN-γ" (right). Gene sets are derived from six cell lines (MCF10A, MDAMB231, BT20, HS578T, MCF7, and SKBR3) provided by Enrichr[27] **c** Proportion of each cell type in CD8+ T cell clusters at uninfected control (n = 3), 2 dpi (n = 3), and 5 dpi (n = 4). Source data are provided as a Source Data file.

Supplementary Fig. 5

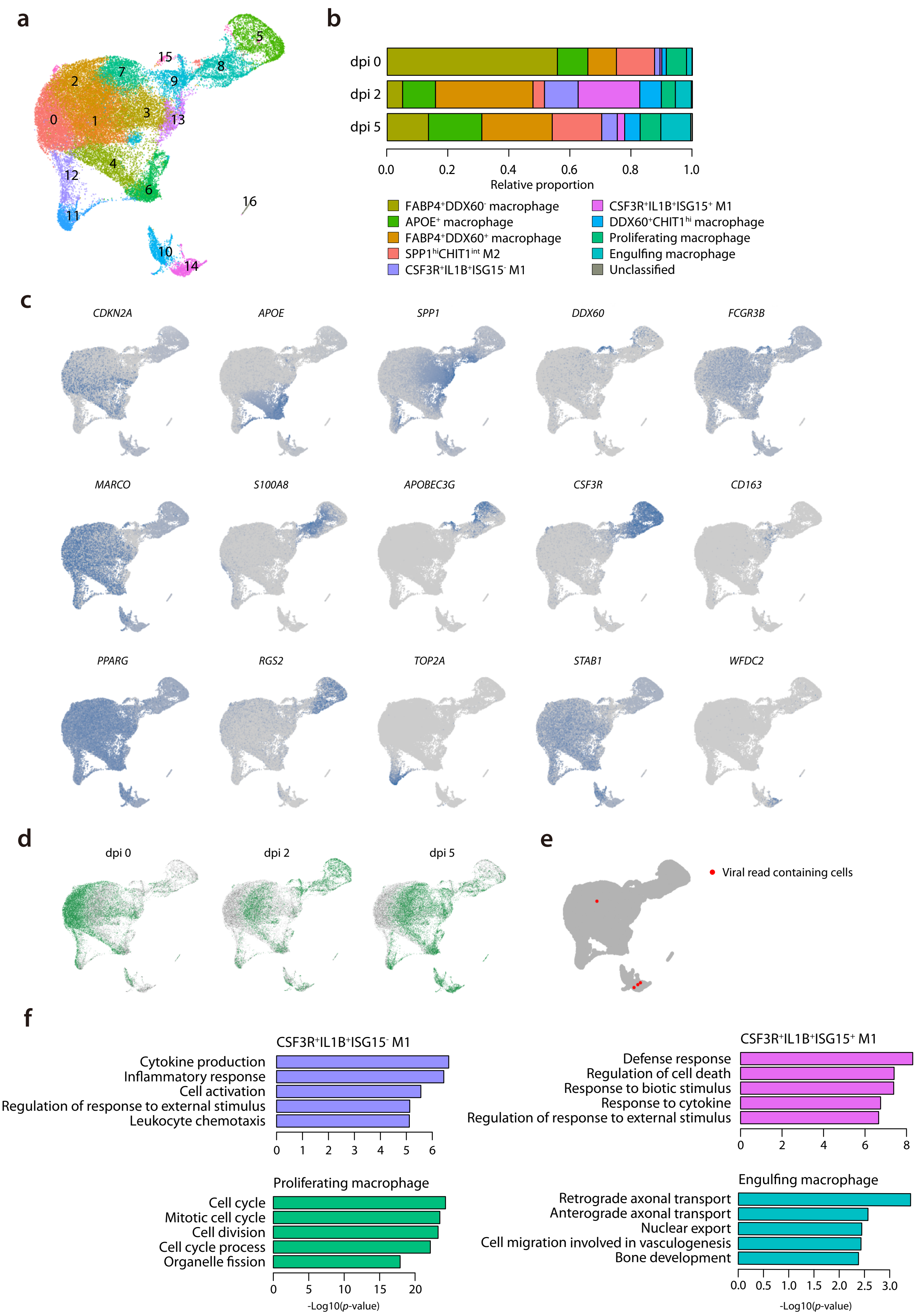

Supplementary Fig. 5 (Cont.)

Supplementary Fig. 5. Sub-clustering of macrophages. **a** UMAP plot of macrophage subpopulations, colored according to clusters. **b** Proportion of each macrophage subpopulation at 0 days post-infection (dpi) (n = 3), 2 dpi (n = 3), and 5 dpi (n = 4). **c** UMAP plots showing normalized expression of known markers of macrophage subpopulations. **d** UMAP plot with color density reflecting distribution of macrophage subpopulations at 0, 2, and 5 dpi. **e** Virus read containing cells (red dot) in UMAP plot of macrophage subpopulations. **f** Bar plots showing –log10(p value) in top-five enrichment analysis of representative GO biological pathways among CSF3R^{+}IL1B^{+}ISG15^{-} (weakly activated M1), CSF3R^{+}IL1B^{+}ISG15^{+} (highly activated M1), proliferating macrophages, and engulfing macrophages. The p values are calculated from a theoretical null-distribution with two-sided Wilconxon signed-rank test. Source data are provided as a Source Data file.

Supplementary Fig. 6

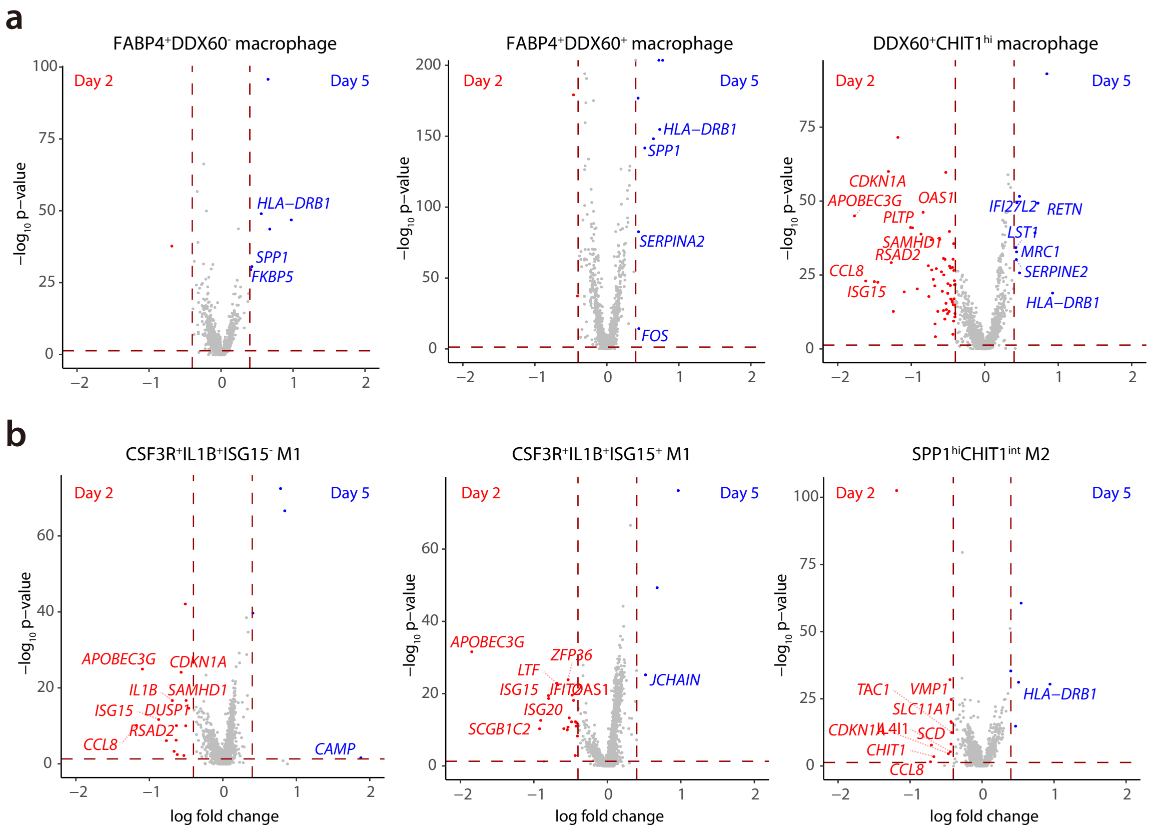

Supplementary Fig. 6. DEGs of macraphages between 2 dpi and 5 dpi. a, b Volcano plots showing DEGs between 2 days post-infection (dpi) and 5 dpi among FABP4$^+$DDX60$^-$ macrophages (resting tissue macrophages), FABP4$^+$DDX60$^+$ macrophages (activated tissue macrophages), DDX60$^+$CHIT1^{hi} macrophages (monocyte-derived infiltrating), CSF3R$^+$IL1B$^+$ISG15$^-$ (weakly activated M1), CSF3R$^+$IL1B$^+$ISG15$^+$ (highly activated M1), and SPP1^{hi}CHIT1^{int} M2 (potentially profibrogenic). Each dot indicates an individual gene. Red indicates a gene that is a significant DEG at 2 dpi, and blue indicates a gene that is a significant DEG at 5 dpi. In the graphs, vertical dashed lines indicate |Log fold change| < 0.4, and horizontal dashed lines indicate $p < 0.05$. The p values are calculated with two-sided Wilconxon signed-rank test.

Supplementary Fig. 7

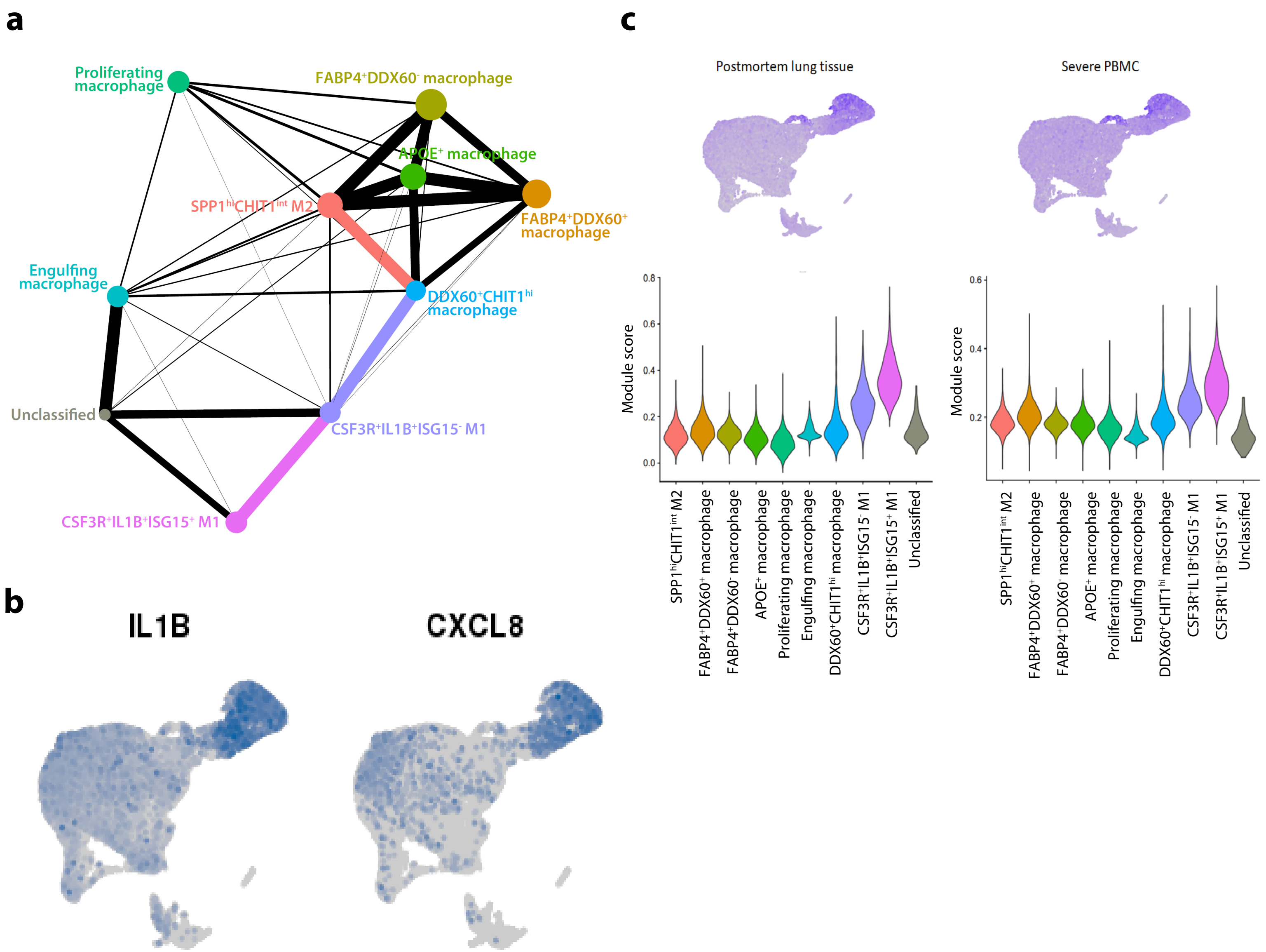

Supplementary Fig. 7. Gene expression patterns of sub-clusters of macrophages. a Partition-based graph abstraction (PAGA) abstracting trajectory analysis among macrophage subpopulations. **b** UMAP plots showing normalized expressions of IL1B and CXCL8 in the macrophage subpopulations. **c** Gene set enrichment analysis of each macrophage subcluster, using publicly available gene sets. Left: Analysis using differentially expressed genes (DEGs) from transcriptome analysis of post-mortem lung tissue from COVID-19 patients and normal lung tissue (GSE147507). Right: Analysis using DEGs from single-cell transcriptome analysis of peripheral blood mononuclear cells (PBMCs) from patients with severe and mild COVID-19 (GSE149689).

Supplementary Fig. 8

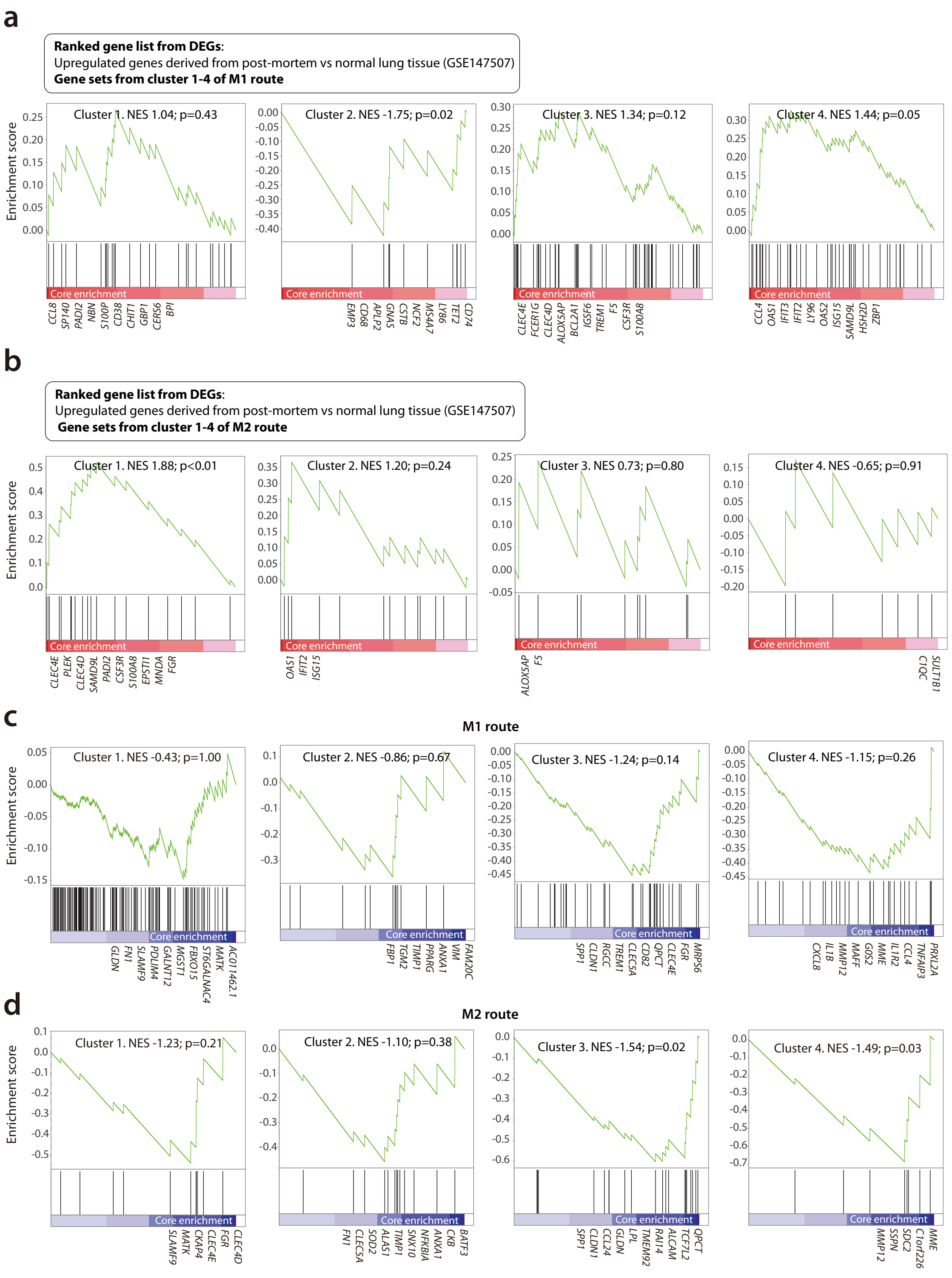

Supplementary Fig. 8. GSEA using DEGs of cluster 1-4 of the M1 and M2 route. a, b GSEA of clusters 1–4 of the M1 route a. and M2 route b. using ranked gene list originated from public transcriptome data, including post-mortem lung tissue from a COVID-19 patient (GSE147507). **c, d** GSEA of clusters 1–4 of the M1 route c. and M2 route d. using ranked gene list originated from etanercept-downregulated DEGs derived from in vitro experiment described in Fig. 5c. The name of genes included as core enrichment were listed, NES, normalized enrichment score. The p values of GSEA is the probability under the null -distribution calculated by permutation test.
