# Supplementary material for: Single-cell transcriptome of bronchoalveolar lavage fluid reveals sequential change of macrophages during SARS-CoV-2 infection in ferrets
Source: Nat Commun. 2021 Jul 27;12:4567. doi: 10.1038/s41467-021-24807-0 (PMC8316405; doi:10.1038/s41467-021-24807-0)
Supplement: Supplementary file 2 — Description of Additional Supplementary Files [file 41467_2021_24807_MOESM2_ESM.pdf]

Description of Additional Supplementary Files

File Name: Supplementary Data 1
Description: List of Marker Genes for Each Cluster of Total BAL Fluid Cells

File Name: Supplementary Data 2
Description: List of Marker Genes for Each Subcluster of NK Cells

File Name: Supplementary Data 3
Description: List of Marker Genes for Each Subcluster of CD8+ 907 T Cells

File Name: Supplementary Data 4
Description: List of Marker Genes for Each Subcluster of Macrophages

File Name: Supplementary Data 5
Description: List of Genes Upregulated in Clusters 1–4 of M1 Route Pseudotime

File Name: Supplementary Data 6
Description: List of Genes Upregulated in Clusters 1–4 of M2 Route Pseudotime

File Name: Supplementary Data 7
Description: List of Genes with Rank, Downregulated by Dexamethasone

File Name: Supplementary Data 8
Description: List of Gene with Rank, Downregulated by Etanercept
